# Determinants of Three-Year Change in Children’s Objectively Measured Sedentary Time

**DOI:** 10.1371/journal.pone.0167826

**Published:** 2016-12-12

**Authors:** Andrew J. Atkin, Louise Foley, Kirsten Corder, Ulf Ekelund, Esther M. F. van Sluijs

**Affiliations:** 1 MRC Epidemiology Unit & UKCRC Centre for Diet and Activity Research (CEDAR), University of Cambridge School of Clinical Medicine, Institute of Metabolic Science, Cambridge Biomedical Campus, Cambridge, United Kingdom; 2 Department of Sports Medicine, Norwegian School of Sports Sciences, Ullevål Stadion, Oslo, Norway; Vanderbilt University, UNITED STATES

## Abstract

**Background:**

Sedentary behaviours (SB) are highly prevalent in young people and may be adversely associated with physical and mental health. Understanding of the modifiable determinants of SB is necessary to inform the design of behaviour change interventions but much of the existing research is cross-sectional and focussed upon screen-based behaviours.

**Purpose:**

To examine the social, psychological and environmental determinants of change in children’s objectively measured sedentary time from age 11 to 14 years.

**Methods:**

Data are from the second (2008) and third (2011) waves of assessment in the Sport, Physical Activity, and Eating Behaviour: Environmental Determinants in Young People (SPEEDY) study, conducted in the county of Norfolk, United Kingdom. Longitudinal data on accelerometer assessed sedentary time were available for 316 (53.5% female, 11.2±0.3 years at baseline) and 264 children after-school and at the weekend respectively. Information on 14 candidate determinants, including school travel mode and electronic media ownership, was self-reported. Change in the proportion of registered time spent sedentary was used as the outcome variable in cross-classified linear regression models, adjusted for age, sex, body mass index and baseline sedentary time. Simple and multiple models were run and interactions with sex explored.

**Results:**

Daily sedentary time increased by 30–40 minutes after-school and at the weekend from baseline to follow-up. Participants who travelled to school by cycle exhibited smaller increases in after-school sedentary time (beta; 95%CI for change in % time spent sedentary: -3.3;-6.7,-0.07). No significant determinants of change in weekend sedentary time were identified.

**Conclusions:**

Time spent sedentary increased during the three-year duration of follow-up but few of the variables examined were significantly associated with changes in sedentary time. Children’s mode of school travel may influence changes in their sedentary time over this period and should be examined further, alongside broader efforts to identify modifiable determinants of SB during childhood.

## Introduction

Public health guidelines in the United Kingdom (UK) and other countries recommend that children and adolescents should limit their engagement in screen-based or overall sedentary behaviour [1–3]. These guidelines draw upon an emerging body of epidemiological evidence indicating that sedentary behaviour, particularly television (TV) viewing, may be an independent risk factor for physical and mental health in this population [4,5]. Whilst interest in this field has expanded rapidly in recent years, much of the existing research remains cross-sectional and reliant upon subjective assessments of behaviour, limiting our ability to make causal inferences [6]. Nonetheless, concern amongst policy-makers and public health scientists over the potentially detrimental effect of sedentary behaviour on health has prompted the development of interventions to modify these behaviours in young people. Review evidence indicates that these programmes have been somewhat effective to date, but effects appear to be small, are often limited to particular subsets of behaviour and long-term evaluations are lacking [7]. Further research to inform the development of behaviour change interventions in this population is required.

Surveillance data indicates that sedentary behaviour is highly prevalent in young people and that participation varies according to a range of socio-demographic factors, including sex, ethnicity and socio-economic position [8–11]. There is also consistent evidence that, whilst moderate to vigorous physical activity decreases, sedentary behaviour increases with age, particularly during the transition from childhood to adolescence [12,13]. These data are valuable in highlighting population groups that might benefit from targeted intervention programmes. However, intervention developers also require information on the *modifiable* determinants of behaviour, to inform the design of intervention strategies [14]. Such factors may operate at the individual, social, environmental and policy-levels, as hypothesised within the ecological model [15]. Although a large number of studies have investigated the factors that influence sedentary behaviour in young people, much of this research has been cross-sectional (correlates) and focussed upon TV viewing or other screen-based behaviours [16,17]. Although such behaviours are prevalent in this population, they are just one subset of sedentary behaviour and only weakly predictive of overall sedentary time [18].

A recent review of longitudinal studies confirmed the previously observed variation in sedentary behaviour at the individual level but highlighted a lack of consistent evidence on modifiable determinants within the social and environmental domains [19]. An accompanying commentary called for further studies utilising objective measures of behaviour and that focussed explicitly on devising and testing determinants that are specific to sedentary behaviour [20]. In a previous analysis in the SPEEDY cohort we identified a small number of home environmental and familial factors that were associated with changes in children’s sedentary time over 1 year (age 10 at baseline) [21]. Building upon this previous work, the aim of the current study was to examine the association of sedentary behaviour specific social, psychological and environmental characteristics with change in children’s objectively measured sedentary time from age 11 to 14 years.

## Methods

### Design and ethics

The Sport, Physical Activity, and Eating Behaviour: Environmental Determinants in Young People (SPEEDY) study is a population based cohort study investigating factors associated with physical activity, sedentary behaviour and diet in children from the county of Norfolk, UK [22]. Ethical approval for each assessment wave was obtained from the University of East Anglia research ethics committee.

### Data collection procedures

The SPEEDY study included three waves of assessment: baseline (T_0_; age 9/10y; April-July 2007), 1-year follow-up (T_1_; age 10/11y; April-July 2008) and 4-year follow-up (T_2_; age 13/14y; April-July 2011). Full details of participant recruitment and procedures for baseline data collection have been reported previously [22]. Participation at T_0_ was prerequisite for recruitment to either of the subsequent waves. Data from waves T_1_ and T_2_ are used in the current study. Wave T_1_ was used as baseline in this analysis due to the inclusion of items related to the determinants of sedentary time in the T_1_ questionnaire that had not been included in the previous wave. As noted above, we have previously explored the social, familial and environmental determinants of change in sedentary time from T_0_ to T_1_ [21]. At T_1_, study information sheets and consent forms were mailed to all 2064 participants from T_0_. Those who consented were mailed an accelerometer, instruction sheet and questionnaire. Participants were asked to wear the accelerometer for one week and to return it by mail, using an addressed, pre-paid envelope. At T_2_ all participants with a valid home address from T_0_ (n = 1964) were mailed information sheets and consent forms. Through local administrative authorities, we ascertained the number of participants attending each secondary school in Norfolk, but our original consent did not allow us to trace individual participants. We presented the study in Year 9 assemblies at secondary schools attended by at least five original participants. Consent forms were returned to the study office by mail. Subsequent measurements were taken at school following similar procedures as at baseline. To increase recruitment, an extra invitation letter was sent home prior to the holiday (July 2011), resulting in an additional 62 participants being assessed by mail, following the same methodology as T_1_.

### Sedentary time measurement

Sedentary time was measured objectively using an Actigraph (GT1M; Pensacola, FL) accelerometer [23,24], set to record at 5-second epochs. Children were instructed to wear the monitor during waking hours for seven days and to remove it while bathing, showering and swimming. Accelerometer data were analysed using a batch processing program (MAHUffe: http://www.mrc-epid.cam.ac.uk/physical-activity-downloads). A count threshold of 100 counts per minute (cpm) was used to define sedentary time [25,26]. Periods of ≥10 minutes of consecutive zero counts [27,28] and days with <500 minutes of recording between 6am–11pm were excluded [28,29]. Two sedentary time outcome variables were derived and analysed separately; (1) after-school (3–11pm, Monday-Friday) and (2) at the weekend (6am– 11pm, Saturday/Sunday). To account for differences in accelerometer wear time between baseline and follow-up, outcome variables were constructed as change in the proportion of time spent sedentary, calculated as follows: [(T_2_ sedentary time/T_2_ wear time)—(T_1_ sedentary time/ T_1_ wear time)]*100. A minimum of 2 days of weekday data and 1 day of weekend data was required for inclusion in the after-school and weekend analyses respectively.

### Exposures

Fourteen determinants were included in the analysis, grouped under the following headings: behavioural, environmental, social, psychological (Table 1). Information on putative determinants was self-reported by children at T_1_ using previously tested items where possible [30,31].

**Table 1:** Description of candidate determinants.

Variable	Description	Summary†(% or mean(SD))	
Behavioural			
School travel mode	Item: How do you usually travel to school? Response options: Car (ref), Bus/Train, Cycle, Walk.	Car	37.2
		Bus/Train	8.3
		Cycle	7.1
		Walk	47.4
After school destination	Item: Do you usually go anywhere else on your way home from school? Response options: Home only (ref), Friend’s house, Shops/Park/Other.	Home	72.4
		Friends house	7.4
		Other	20.2
Environmental			
Home location	Home located in rural (ref) / urban location. Derived from home postcode using methods described by Bibby and Shephard [44]. Four density profiles were collapsed: ‘city’/‘town and fringe’ classified as Urban, ‘hamlets and isolated dwellings’/‘villages’ classified as Rural.	Urban	37.1
		Rural	62.9
Car ownership	Item: Which of the following things do you have at home? Prompt: More than 1 car. Response options: No (ref), Yes.	No	34.3
		Yes	65.7
Home—games console	Item: Which of the following things do you have at home? Prompt: A games console. Response options: No (ref), Yes.	No	20.7
		Yes	79.3
Home—active games console	Item: Which of the following things do you have at home? Prompt: An active games console. Response options: No (ref), Yes.	No	54.2
		Yes	45.8
Bedroom—electronic media	Number of electronic media items present in participant’s bedroom: television, DVD/video player, personal computer, video games console. Sum score: range 0–4.		1.5 (1.3)
Bedroom—active games console	Item: Which of these do you have in your bedroom? Prompt: An active games console. Response options: No (ref), Yes.	No	92.6
		Yes	7.4
Social			
School travel—social context	Item: Who do you usually travel to school with? Response options: Accompanied by an adult (ref), Alone, Friend/Sibling.	Adult	45.5
		Alone	5.8
		Friend/Sibling	48.7
Social support for limiting SB*	Seven items indicating social and environmental restrictions on SB. Example items: ‘My parents tell me to watch less TV’ (reverse coded), ‘I can play a computer game for as long as I want’. Response options: Never (coded 2), Sometimes (coded 1), Always (coded 0). Sum score: range 0–14. Cronbach’s alpha: 0.4.		7.5 (1.8)
Psychological			
Negative perception of SB*	Four items indicating participant’s negative perception of screen-based SB. Example items: ‘I think TV and video games are boring’, ‘Watching TV takes time away from other fun activities’. Response options: Yes (coded 2), Don’t know (coded 1), No (coded 0). Sum score: range 0–8. Cronbach’s alpha: 0.4.		4.3 (1.8)
Positive perception of SB*	Four items indicating participant’s positive perception of screen-based SB. Example items: ‘I enjoy playing computer games for many hours in a row’, ‘Watching TV is my favourite pastime’. Response options: Yes (coded 2), Don’t know (coded 1), No (coded 0). Sum score: range 0–8. Cronbach’s alpha: 0.4.		3.3 (1.8)
Self-efficacy for SB change*	Five items assessing perceived ability to limit SB. Example items: ‘I can turn off the TV even when there is a programme on that I enjoy’, ‘I can leave the room when others are watching TV’. Response options: Yes (coded 2), Don’t know (coded 1), No (coded 0). Sum score: range 0–10. Cronbach’s alpha: 0.4.		7.9 (1.6)
Enjoyment of SB	Item: I enjoy sedentary activities. Response options: No (ref), Don’t know, Yes.	No	26.1
		Don’t know	30.1
		Yes	43.8

SD, standard deviation; SB, sedentary behaviour; ref, reference group.

^†^ Summary data are provided for participants included in the after-school analysis.

* Items were adapted from questionnaires developed previously by Norman et al. [30,31].

### Covariates

Date of birth and sex were self-reported. Height and weight were measured by trained research assistants at baseline (T_0_) and used to calculate body mass index (BMI, kg/m2).

### Statistical analysis

Analyses were conducted using Stata (version 13.0) in 2015. We compared demographic characteristics and responses to exposure variables between those included in the analysis and those lost to follow-up using Student’s t tests and chi-squared tests. Cross-classified linear regression models were used to examine associations between candidate determinants (assessed at T_1_) and changes in the proportion of time spent sedentary after-school and at the weekend. The cross-classified model accounts for the clustering of participants within primary (T_1_) and secondary (T_2_) schools but does not assume a hierarchical structure. This is because children from any given primary school attended several different secondary schools, and each secondary school received pupils from several different primary schools. Initially, associations between determinants and sedentary time outcomes were examined with adjustment for age, sex, BMI and baseline level of the outcome variable (simple models) [32]. Determinants associated at P<0.1 were retained for inclusion in a multivariable model [21]. Subsequently, interaction terms were added to simple regression models to examine effect modification by sex. Interactions were retained for inclusion in the multivariable model where they met the following criteria: 1) the interaction term P-value was <0.1; and 2) the determinant was associated at P<0.05 in either boys or girls. This strategy was employed to simplify interpretation of the final multivariable model and to reduce the risk of type 1 error resulting from multiple hypothesis testing. In the multivariable model, statistical significance was set at P<0.05.

## Results

Data for the current analysis are from the second (T_1_) and third (T_2_) waves of assessment in the SPEEDY study. Of the 2064 children invited to participate at T_1_, 1019 (49.4% of invited) obtained parental consent and took part in assessments. Three years later, 1964 children who had valid contact details at T_1_ were invited to participate in the final wave of assessment (T_2_), of which 480 (24.4% of invited) obtained consent. Valid accelerometer data on changes in after-school and weekend sedentary time were obtained from 316 and 264 participants respectively. Participants included in the after-school analysis were more likely to have a mother who remained in education beyond 16 years of age (47.0% vs. 37.3%, p = 0.01) and less likely to live in rented accommodation (15.2% vs. 25.1%, p<0.01) than those who took part at T_1_ but did not provide valid after-school outcome data. There were, however, no differences in age or sex between the analytical sample and those lost to follow-up. Participants included in the after-school analysis were less likely to travel home from school alone (5.8% vs. 11.4%, p = 0.02), less likely to own a video games console (79.3% vs. 84.6%, p = 0.04), had fewer items of electronic media in the bedroom (1.5. vs. 1.9 item, p<0.01) and had higher levels of social support for limiting their sedentary behaviour (score 7.5 vs. 7.2, p = 0.03) than those who took part at T_1_ but did not provide valid after-school outcome data. Demographic characteristics of the 316 participants that provided valid data on after-school sedentary time at T_1_ and T_2_ are reported in Table 2. Over 3 years, daily sedentary time increased by approximately 30–40 minutes after-school and at the weekend (Table 3).

**Table 2:** Baseline characteristics of participants with valid after-school accelerometer data at baseline and follow-up.

	All	Girls	Boys
**Sex, n (%)**	316	169 (53.5)	147 (46.5)
**Age, mean(SD)**	11.2 (0.3)	11.2 (0.3)	11.2 (0.3)
**Maternal education, n (%)**			
Left education <16y	115 (37.3)	69 (41.3)	46 (32.6)
Left education >16y	193 (62.7)	98 (58.7)	95 (67.4)
**House tenure, n (%)**			
Renting	47 (15.2)	28 (16.7)	19 (13.5)
Buying	262 (84.8)	140 (83.3)	122 (86.5)

SD, standard deviation.

Maternal education: All n = 308, boys n = 141, girls n = 167.

House tenure: All n = 309, boys n = 141, girls n = 168.

**Table 3:** Accelerometer wear time and sedentary time after-school and at the weekend. Mean (SD).

	After-school^a^			Weekend		
	T_1_	T_2_	Change	T_1_	T_2_	Change
**Sedentary time,(min/day)**						
All	206.1 (41.2)	242.4 (48.1)	+36.4 (55.5)	444.6 (71.1)	482.7 (79.6)	+38.1 (89.4)
Girls	210.8 (40.7)	243.7 (44.0)	+32.8 (53.1)	445.3 (67.2)	476.6 (69.7)	+31.3 (86.9)
Boys	200.5 (41.2)	241.0 (52.5)	+40.4 (58.1)	443.8 (75.4)	489.3 (88.9)	+45.5 (91.7)
**Wear time,(min/day)**						
All	323.8 (55.4)	341.8 (58.5)	+18.1 (69.3)	691.0 (79.7)	682.1 (93.4)	-9.0 (112.9)
Girls	327.8 (55.0)	339.8 (54.3)	+12.0 (65.9)	691.8 (75.0)	672.3 (88.0)	-19.5 (107.8)
Boys	319.1 (55.7)	344.2 (63.0)	+24.0 (72.8)	690.2 (84.8)	692.6 (98.2)	+2.4 (117.5)
**Sedentary time**, **% of wear time**						
All	63.7 (6.8)	70.9 (7.1)	+7.2 (8.4)	64.4 (7.8)	70.9 (8.2)	+6.6 (9.1)
Girls	64.4 (6.7)	71.7 (6.4)	+7.3 (7.8)	64.4 (7.3)	70.8 (9.1)	+6.7 (8.5)
Boys	62.9 (6.9)	69.9 (7.7)	+7.0 (9.2)	64.3 (8.3)	71.1 (7.3)	+6.4 (9.8)

SD, standard deviation.

^a^ After-school defined as 3–11pm Monday to Friday.

After-school: All n = 316, boys n = 147, girls n = 169.

Weekend: All n = 264, boys n = 127, girls n = 137.

Simple associations between candidate determinants and changes in the proportion of time spent sedentary after-school and at the weekend are presented in Table 4. Three variables were associated with change in after-school sedentary time in simple models and carried over for inclusion in a multivariable model (Table 5). One variable remained significant in the multivariable model. Participants who reported that they travelled to school by cycle exhibited smaller increases in their after-school sedentary time. The presence of an active games console in the home (p = 0.068) was positively associated with change in weekend sedentary time. No other exposures were associated with change in weekend sedentary time. A multivariable model for change in weekend sedentary time, therefore, was not required.

**Table 4:** Simple associations of behavioural, environmental, social and psychological factors with changes in after-school and weekend sedentary time.

Variable	After-school^a^		Weekend	
	β	95% CI	β	95% CI
Behavioural				
School travel mode				
Car (ref)				
Bus/Train	0.82	(-2.1, 3.7)	1.5	(-2.0, 5.0)
Cycle	**-3.4**	(-6.6, -0.20)**	-1.4	(-5.2, 2.5)
Walk	-0.53	(-2.2, 1.1)	0.81	(-1.3, 2.9)
After school destination				
Home (ref)				
Friends house	1.3	(-1.8, 4.4)	0.28	(-3.4, 3.9)
Park/Shops/Other	0.52	(-1.5, 2.5)	-0.57	(-3.0, 1.8)
Environmental				
Home location	-0.70	(-2.3, 0.94)	0.60	(-1.6, 2.8)
Car ownership	0.62	(-1.0, 2.3)	-1.3	(-3.3, 0.67)
Home—games console	0.90	(-1.0, 2.8)	-0.91	(-3.2, 1.4)
Home—active games console	-0.89	(-2.4, 0.65)	**1.7**	(-0.13, 3.5)*
Bedroom—electronic media	0.0026	(-0.62, 0.62)	0.16	(-0.59, 0.91)
Bedroom—active games console	1.3	(-1.6, 4.2)	2.4	(-0.95, 5.8)
Social				
School travelȁsocial context				
Accompanied by an adult (ref)				
Alone	0.11	(-3.2, 3.4)	-0.63	(-4.5, 3.3)
Friend/Sibling	0.37	(-1.2, 2.0)	0.73	(-1.2, 2.7)
Social support for limiting SB	-0.16	(-0.59, 0.27)	0.34	(-0.20, 0.89)
Psychological				
Negative perception of SB	-0.12	(-0.56, 0.31)	0.00044	(-0.53, 0.54)
Positive perception of SB	**0.25**	**(-0.19, 0.70)**†	-0.082	(-0.63, 0.47)
Self-efficacy for SB change	0.08	(-0.39, 0.55)	-0.27	(-0.85, 0.31)
Enjoyment of SB				
No (ref)				
Don’t know	0.79	(-1.3, 2.9)	1.0	(-1.5, 3.6)
Yes	**2.0**	(0.15, 3.9)**	0.69	(-1.7, 3.0)

β, beta coefficient; 95% CI, 95% confidence interval; ref, reference group.

* p<0.1;

** p<0.05;

^†^ evidence of interaction with sex.

^a^ After-school defined as 3–11pm Monday to Friday.

Models adjusted for: age (continuous), sex (male, female), body mass index (continuous).

After-school (n = 289–306) and weekend (n = 238–251) analytical samples vary due to missing data for individual determinants.

**Table 5:** Final multivariable model for association of candidate determinants with change in the proportion of after-school time spent sedentary.

Variable	β	95% CI
School travel mode		
Car (ref)		
Bus/Train	1.2	(-1.7, 4.1)
Cycle	**-3.3**	(-6.5, -0.07)*
Walk	-0.18	(-1.9, 1.5)
Positive perception of SB (boys)^a^	0.63	(-0.08, 1.3)
*Sex interaction*	-0.85	(-1.7, 0.04)
Enjoyment of SB		
No (ref)		
Don’t know	0.29	(-1.8, 2.4)
Yes	1.6	(-0.39, 3.6)

β, beta coefficient; 95% CI, 95% confidence interval; SB, sedentary behaviour; ref, reference group.

* p<0.05,

** p<0.01.

^a^ β (95% CI) for association in girls: -0.22 (-0.82, 0.37).

After-school defined as 3–11pm Monday to Friday.

Models adjusted for: age (continuous), sex (male, female), body mass index (continuous) and mutually adjusted for named determinants.

## Discussion

Between the ages of 11 and 14 years, participants in the current study increased their daily sedentary time by approximately 30–40 minutes after-school and at the weekend. Overall, few of the candidate determinants examined were significantly associated with these changes in sedentary time, but participants that used a bicycle for their journey to school showed smaller increases in sedentary time over three years. No significant determinants of change in weekend sedentary time were observed.

Over the three-year follow-up period, travelling by cycle for the journey to school was associated with a smaller increase in children’s overall sedentary time after school. Relative to those who travelled by car and assuming monitor wear time of 12 hours/day, use of a cycle for the journey to school was associated with an approximately 24 minute smaller increase in sedentary time. That is, where the sample mean change in sedentary time after-school was an increase of approximately 36 minutes/day, travel by cycle to school was associated with an increase of approximately 12 minutes/day. To our knowledge, the role of school travel as a determinant of change in children’s sedentary time has not been examined previously. There is evidence that active school travel is associated with higher levels of physical activity, though associations are typically stronger and more consistent for walking than cycling [33–35]. This may partially be a reflection of the generally low prevalence of cycling to school and the methodological challenges associated with assessing cycling-based activity. Given the paucity of research in this area and the associated methodological challenges, our findings warrant further investigation but should be interpreted cautiously at present.

In the simple regression model, boys who expressed a stronger positive attitude towards sedentary behaviour exhibited larger increases in after-school sedentary time, but this association was attenuated, and no longer significant, in the multivariable model. Given the strict criteria we employed for the exploration of interactions, the role of attitudes in shaping children’s sedentary behaviour, the assessment thereof and possible effect modification by sex, may be worthy of further investigation. This would be most valuable in large population based cohorts with sufficient participants to enable exploration of interactions. It is unclear why the observed trend was limited to boys, but it is possible that the items used to assess attitudes towards sedentary behaviour better captured this construct in boys than girls. Individual items focussed specifically upon TV viewing and computer use, for example, which are typically more prevalent in boys than girls [10,18]. Previous longitudinal research examining the association of children’s attitudes with their overall sedentary time is lacking and evidence linking attitudes with screen-based sedentary behaviour and physical activity in this population is mixed [16,17,36]. Our findings indicate that exposure measurement tools should be developed with consideration to possible differential functioning between sexes and that statistical testing for interactions with sex should be included in future studies exploring the determinants of sedentary time.

No significant determinants of change in weekend sedentary time were identified. There was a trend for participants that reported having an active games console in the home to exhibit larger increases in sedentary time but the association did not reach our a priori determined level of significance (p<0.05). Despite the intuitive appeal, none of the four exposures related to the home media environment were strongly associated with changes in sedentary time. Previous experimental work has indicated that the introduction of an active games console into the home may reduce passive gaming, increase physical activity and reduce overall sedentary time, but results are inconsistent and effects appear to wane over time [37,38]. Interestingly, a recent observational study in Irish children (age 12 years) noted that active games consoles were used predominantly to play passive games and that active video game play was positively correlated with TV viewing [39]. These complex interrelations between electronic media and sedentary behaviour in children suggest that interventions that focus solely upon the provision or removal of electronic devices from the home may be overly simplistic and unlikely to produce consistent changes in behaviour. Indeed, qualitative studies have shown that diverse features of the home social and physical environment interact to influence children’s physical activity and sedentary behaviour [40,41]. Researchers and practitioners will need to acknowledge these issues during intervention design. It may, for example, be necessary to enable some degree of family-level tailoring of intervention content to account for the unique characteristics of individual homes.

A total of 14 exposure variables from the behavioural, environmental, social and psychological domains were examined for their association with sedentary time in the current study, but few significant associations were identified. The reasons for this are unclear but a number of explanations may be hypothesised. Firstly, it may be that changes in discretionary sedentary time are driven by a small number of key variables. Whilst this is plausible, few candidates have emerged from the research conducted to date, which is characterised by considerable inconsistency between studies [19]. An alternative explanation is that there was a lack of specificity between exposure and outcome variables used in this analysis [42]. Differential associations between candidate exposures and individual sedentary behaviours may result in associations being attenuated to the null when an aggregate-level outcome (i.e. total sedentary time) is used. We attempted to address this limitation by examining after-school and weekend behaviour separately but this may not have been sufficient. Continued effort to develop objective measures of individual sedentary behaviours is necessary. Consistent with a recent call in the literature, the exposures used in this analysis were devised specifically for the exploration of sedentary behaviour [20]. Nonetheless, further development and testing may be required to refine and optimise these items, particularly those related to social or psychological constructs.

A key strength of this study is the use of accelerometry to obtain objective assessments of sedentary time in a population based cohort of children. This analysis adds to a small body of longitudinal evidence examining the determinants of change in children’s sedentary time. Exposure variables were derived for multiple levels of the ecological model and statistical analyses accounted for the clustering of participants in primary and secondary schools at baseline and follow-up. Multivariable models were constructed to control for confounding and to identify independent associations amongst exposure variables where appropriate. The following limitations are acknowledged. Firstly, although our analytical strategy accounted for a number of potential confounders, we were not able to adjust for sexual maturation, which may have resulted in residual confounding. In addition, the analytical sample was relatively small due to participant attrition between baseline and follow-up assessments. Although sample retention was similar to that in other cohorts in this population [43] it is possible that some of our analyses were underpowered. However, most regression estimates were small, suggesting that the predominantly null findings cannot be attributed solely to a lack of statistical power. There was evidence that participants from families of lower socio-economic position were more likely to drop-out and that the analytical sample generally lived in homes that had fewer electronic media or were more supportive of limiting sedentary behaviour, potentially limiting the generalisability of our findings. Some of our social and environmental exposures were assessed with items that pertained to screen-based behaviours specifically, rather than overall sedentary time, which may have limited their predictive capacity. In addition, the internal consistency of composite exposure measures was lower than ideal, likely due to our use of a limited range of response options intended to maximise participant comprehension. We recognise the need for further development of these tools, but contend that there inclusion is justified given the exploratory nature of the analysis and limited available evidence on this topic. Lastly, data used in this analysis were collected in 2008 / 2011 and it is possible that behaviour patterns and exposure characteristics may have changed since this time. It is unknown, however, whether exposure-outcome associations changed commensurately over this period.

### Conclusion

In this population-based cohort of English children, sedentary time increased by 30–40 minutes per day between the ages of 11 and 14 years. Few of the exposure variables examined were predictive of changes in sedentary time, though the role of school travel mode as a determinant of change in children’s sedentary behaviour is worthy of additional examination. Further work to identify the modifiable determinants of change in sedentary behaviour during the transition from childhood to adolescence is required. Methodological developments related to the assessment of sedentary behaviour specific exposures and outcomes may be beneficial to this process.

## Supporting Information

S1 File Data file. (XLSX)
